# Blockade of P2X7 receptors preserves blood retinal barrier integrity by modulating the plasmalemma vesicle-associated protein: Implications for diabetic retinopathy

**DOI:** 10.1111/bph.70007

**Published:** 2025-02-20

**Authors:** Chiara Bianca Maria Platania, Francesca Lazzara, Kenneth Mitton, Naomi Haque, Wendelin Dailey, Federica Conti, Erika Giuffrida, Filippo Drago, Anca Hermenean, Cornel Balta, Hildegard Herman, Alina Ciceu, Maria Consiglia Trotta, Michele D’Amico, Giuseppe Nicosia, Settimio Rossi, Claudio Bucolo

**Affiliations:** 1 Department of Biomedical and Biotechnological Sciences, School of Medicine, University of Catania, Catania, Italy; 2 Center for Research in Ocular Pharmacology—CERFO, University of Catania, Catania, Italy; 3 Eye Research Institute, Oakland University, Rochester, Michigan, USA; 4 “Aurel Ardelean” Institute of Life Sciences, Vasile Goldis Western University of Arad, Arad, Romania; 5 Department of Experimental Medicine, Division of Pharmacology, University of Campania “Luigi Vanvitelli”, Naples, Italy; 6 Eye Clinic, Multidisciplinary Department of Medical, Surgical and Dental Sciences, University of Campania “Luigi Vanvitelli”, Naples, Italy

**Keywords:** blood retinal barrier, choroid, diabetic retinopathy, P2X7 receptor, PLVAP, VE-cadherin, ZO-1

## Abstract

**Background and Purpose:**

Plasmalemma vesicle-associated protein (PLVAP) regulates transcytosis in vascular endothelial cells. PLVAP expression is increased in pathological conditions, such as diabetic retinopathy. P2X7 receptor antagonists have been shown to preserve blood-retinal barrier (BRB) integrity. Here, we have tested the hypothesis that PLVAP expression is tightly linked to P2X7 receptor activity, leading to breakdown of the BRB in an in vitro model of diabetic retinopathy.

**Experimental Approach:**

We integrated network approaches with an in vitro model of diabetic retinopathy using primary human retinal microvascular endothelial cells (HRMECs). Cells were treated with a P2X7 receptor antagonist, JNJ47965567, and expression of several genes predicted to belong to the P2X7 receptor signalling network were assessed. Levels and localisation of PLVAP, VE-cadherin and zonula occludens-1 (ZO-1) in HRMECs were evaluated. In vivo, the effects of JNJ47965567 on PLVAP expression in the retinas of diabetic mice were assessed.

**Key Results:**

High levels of glucose increased PLVAP expression in HRMECs, which was blocked by JNJ47965567. Furthermore, JNJ47965567 preserved VE-cadherin and ZO-1. In the choroidal vasculature of diabetic mice, PLVAP immunostaining was increased, compared to levels in non-diabetic mice. This increase was significantly attenuated by treatment with JNJ47965567

**Conclusions and Implications:**

This study showed that P2X7 receptor signalling is an important component of a complex gene regulatory network, including PLVAP, mediating the pathophysiology of diabetic retinopathy. The P2X7 receptor antagonist JNJ47965567 showed a good pharmacodynamic profile, suggesting that this approach could be of value in the treatment of diabetic retinopathy.

## 1 | INTRODUCTION

Diabetic retinopathy is a microvascular complication of diabetes mellitus. About 5–8% of diabetic subjects develop diabetic retinopathy (Lee et al., 2015). Therefore, given the increased incidence of diabetes in developed countries, the overall incidence of diabetic retinopathy will increase dramatically. The socio-economic impact of diabetic retinopathy is huge, mainly because of consequences related to irreversible blindness, caused by diabetic macular oedema, and related retinal detachment. Currently, pharmacological interventions of diabetic macular oedema are intravitreal injections of steroids and anti-VEGF-A agents (Bonfiglio et al., 2020; Bucolo et al., 2018), along with laser therapy (Bonfiglio et al., 2022). Intravitreal injections are not only expensive but also associated to poor compliance and in some cases may not be effective in non-responsive subjects. Given these unmet medical needs of diabetic retinopathy pharmacological management, more efforts must be made to find new and effective therapeutics for diabetic retinopathy.

In this context, the purinergic P2X7 receptor is emerging as an interesting pharmacological target for the treatment of diabetic retinopathy and other retinal diseases (Di Virgilio et al., 2023; Platania et al., 2022; Tassetto et al., 2021). Antagonists of P2X7 receptors exerted anti-inflammatory effects in preclinical in vitro and in vivo models of diabetic retinopathy, and protected the blood retinal barrier (BRB) from hyperglycaemia induced damage. Specifically, P2X7 receptor antagonism promoted the expression of adherens and tight junctions, thus maintaining the integrity of the BRB by inhibition of paracellular transport (Platania et al., 2019). In addition to the regulation of BRB integrity, activation of P2X7 receptors in microglia and macrophages is a trigger of retinal inflammation, involving also CD40-TRAF2.3 signalling in Müller cells (Portillo et al., 2022). Regarding transcellular transport, the plasmalemma vesicle-associated protein (PLVAP) affects barrier integrity through caveolae formation and fusion into trans-endothelial channels, along with endothelial fenestrae (Bosma et al., 2018; Denzer et al., 2023). PLVAP expression in the mouse neural retina is initially high during formation of the neural retina’s vasculature and is rapidly decreased as the BRB matures. Thereafter, up-regulation of PLVAP is involved in several pathological conditions including the loss of normal BRB integrity in retinal vascular diseases (Van Der Wijk et al., 2019). The Norrin–Wnt signalling pathway is required for the developmental repression of endothelial PLVAP expression in the mouse retina (Zhang et al., 2018) and dysfunctional variants of several proteins in the canonical Norrin–Wnt pathway cause familial exudative vitreous retinopathy in humans (Le et al., 2023). Interestingly, genetic variants of PLVAP are associated with rare and severe protein-losing enteropathies, because of a compromised endothelial barrier function, as reported in several case studies (Gorukmez et al., 2019; Kurolap et al., 2018).

Blood brain barrier (BBB) function inversely correlates with PLVAP expression (Schlingemann et al., 1988). In fact, while PLVAP is necessary for the development of the BRB (Van Der Wijk et al., 2019), this protein is not highly expressed in the mature BRB, and PLVAP immunostaining was increased in post-mortem retinal samples of diabetic patients, compared to control (Schlingemann et al., 1999; Witmer et al., 2002). Other studies reported an increased expression of PLVAP in monkeys where retinopathy was induced by intravitreal injections of VEGF-A (Hofman et al., 2001; Witmer et al., 2002). Additionally, Nakagami et al., (2019) have shown that PLVAP expression is induced in the retina of rat models of retinopathy of prematurity (ROP). In the same study, authors confirmed that intravitreal administration of anti-PLVAP (polyclonal, 20–100 μg in 50 μl) suppressed laser-induced choroidal neovascularisation in monkeys (Nakagami et al., 2019).

Based on these results, we explored, in an in vitro model of BRB (endothelial cell monolayer), the effects of P2X7 receptor inhibition on a predicted gene–gene network, which was enriched on the basis of experimental data obtained in the present study. Further, we investigated the effects of inhibiting P2X7 receptors on the regulation of PLVAP expression in vivo, through intravitreal injection of JNJ47965567, in diabetic CD1 mice.

## 2 | METHODS

### 2.1 | Network analysis

We accessed the ‘multiple protein’ search in the webserver STRING (Szklarczyk et al., 2015). An enriched network was built with STRING application implemented within the Cytoscape software v. 3.7.0 (Montojo et al., 2010). Centrality metrics and topology analyses were carried out within Cytoscape environment. Centrality metrics and topology analyses were plotted in the network, and network images have been built with Cytoscape.

### 2.2 | Cell culture

As human choroidal endothelial cells are supplied by very few vendors, and they are not well characterised in published papers, we used primary human retinal microvascular endothelial cells (HRMECs), which were purchased from Innoprot (Elexalde Derio, Spain, Cat# P10880, RRID: CVCL_B5WJ) and from Cell Systems (Kirkland, WA). This cell line was cultured at 37°C in a humidified atmosphere of 5% CO_2_, in Innoprot-formulated Endothelial Medium (Cat# P10880) with 100 U ml^−1^ penicillin, 100 μg ml^−1^ streptomycin, 1% endothelial cell supplement factor (ECGS) and 5% foetal bovine serum. At Oakland University, HRMECs were cultured in EndoGRO Basal Medium (Cat# SCME004, Millipore) prepared with growth supplements according to the manufacturer’s instructions for a final concentration of 0.2% low serum supplement, 5 ng ml^−1^ epidermal growth factor, 50 μg ml^−1^ ascorbic acid, 10 mM L-Glutamine, 1.0 μg ml^−1^ hydrocortisone hemisuccinate, 0.75 U ml^−1^ heparin sulphate and 2% foetal bovine serum (Millipore, Burlington, MA). For all experiments, HRMEC cells (passage from 2 up to 5) were seeded and after reaching confluence (80%) were grown in 5-mM glucose medium (Ctrl group cells); shifted to high glucose medium for 48 h (40 mM, HG group cells); pre-treated for 1 h with 100 nM of the P2X7 receptor antagonist JNJ47965567, and with high glucose for 48 h (40 mM) (JNJ + HG group cells), as already reported (Lazzara et al., 2019; Platania et al., 2019). The concentration (100 nM) of JNJ47965567 was chosen on the basis of pharmacodynamic properties on P2X7 receptor channel activity as reported by Bhattacharya et al. (2013). In our experiments, 100 nM JNJ47965567 led within 6 min to steady state P2X7 receptor channel blocking. Additionally, 100 nM JNJ47965567 has been shown to be effective in our in vitro models of diabetic retinopathy (Fresta, Caruso, et al., 2020; Platania et al., 2017, 2019).

### 2.3 | Extraction of total ribonucleic acid and cDNA synthesis and quantitative real-time polymerase chain reaction (qRT-PCR)

Extraction of total RNA, from HRMEC cells was performed, after 48 h of treatment, with a TRIzol Reagent (Invitrogen, Life Technologies, Carlsbad, CA, United States) and by using Monarch Total RNA Miniprep Kit according to the cultured mammalian cells instructions (New England Biolabs, Ipswich, MA). RNA concentration was determined by the A_260/280_ ratio with Multimode Reader Flash Varioskan^™^ and Eppendorf Bio-Spec; the RNA purity was confirmed with the electrophoresis in non-denaturing 1% agarose gel (in TAE). cDNA was synthesised from 2 or 1 μg of total RNA with a reverse transcription kit (SuperScript^™^ II Reverse transcriptase, Invitrogen, Thermo Fisher Scientific, Carlsbad, CA, USA) or with the LunaScript RT Super Mix Kit (New England Biolabs, Ipswich, MA), respectively.

SYBR Green real-time PCR was carried out with the Rotor-Gene Q (Qiagen). The amplification reaction mix included the Master Mix Qiagen (10 μl) (Qiagen QuantiNova SYBR Green Real-Time PCR Kit) and cDNA (1 μl, 100 ng). Forty-five amplification cycles were carried out for each sample. Results were analysed with the 2^−ΔΔCt^ method. Quantitative PCR experiments followed the MIQE guidelines (Bustin et al., 2009). Gene expression levels were normalised with levels of housekeeping gene (18S). Primers were purchased from Eurofins Genomics (Milan, Italy). Forward and reverse primer sequences are herein listed: 18S (forward 5′-AGTCCCTGCCCTTTG-3′; reverse 5′-GATCCGAGGGCCTCACTAAAC-3′), IL-1β (forward: 5′-AGCTACGAATCTCCGACCAC-3′; reverse: 5′-CGTTATCCCATGTGTCGAAGAA-3′), VEGF-A (forward 5′-AGGGCAGAATCATCACGAAG-3′; reverse 5′-ATCCGCATAATCTGCATGGT-3′), PIGF (forward: 5′-ATGTTCAGCCCATCCTGTGT-3′; reverse: 5′-CTTCATCTTCTCCCGCAGAG-3′), P2X7 receptor (forward 5′-AAGCTGTACCAGCGGAAAGA-3′; reverse 5′-GCTCTTGGCCTTCTGTTTTG-3′), Cx43 (forward 5′-GAGTTTGCCTAAGGCGCTC-3′; reverse 5′-AGGAGTTCAATCACTTGGCG-3′), ZO-1 (forward: 5′-CAGCCGGTCACGATCTCCT-3′; reverse: 5′-TCCGGAGACTGCCATTGC-3′), CHD5 (VE-Cadherin) (forward: 5′-GGCAAGATCAAGTCAAGCGTG-3′; reverse: 5′-ACGTCTCCTGTCTCTGCATCG-3′).

TaqMan qPCR assays were performed in triplicate using the Luna Universal Probe qPCR Master Mix, which contains a Rox reference dye (New England Biolabs, Ipswich, MA). A duplex qPCR reaction format was used, consisting of FAM-labelled *PLVAP* TaqMan probe (Hs00229941_m1, spans exons 3–4), TSPAN 12 (Hs01113125_m1, spans exons 7–8), LRP5 (Hs01124561_m1, spans exons 14–15) and *TBP* (*Tata-Binding Protein*) VIC-labelled, primer-limited, TaqMan probe (Hs00427620_m1, spans exons 2–3) as the normaliser gene (Thermo Fisher, Waltham, MA). Triplicate reactions were used for assays of each sample. Reactions were run on AriaMx Real-time PCR System using the AriaMx HRM QPCR Software (Agilent, Santa Clara, CA). Each replicate was normalised to endogenous *TBP* gene expression and evaluated using the delta–delta Ct method. All assays were evaluated for adequate PCR efficiency using a serial dilution series of cDNA. qPCR analysis complied with the MIQE guidelines (Bustin et al., 2009).

### 2.4 | In-cell Western analysis

HRMECs were seeded and grown to confluence with in black-walled 96-well culture plates and reduced to hydrocortisone-free medium 1 day before treatment. Cells were treated with base media (containing 1% FBS, no hydrocortisone, 5 mM glucose), high glucose media (base adjusted to 40 mM glucose), or high glucose with 100 nM JNJ. For this latter group of cells, we carried out JNJ pre-treatment 1 h before shifting to high glucose medium. After 48 h, the cells were fixed with 4% paraformaldehyde for 20 min at room temperature followed by permeabilisation with 0.1% Triton X-100. Cells were then blocked with Odyssey Blocking Buffer (Li-Cor, Lincoln, NE, USA) for 1.5 h at room temperature and then incubated with rabbit IgG anti-PLVAP (NovusBio, Cat# NBP1–83911, RRID:AB_11029033, CO, USA, 1:200 dilution in 50% Odyssey Blocker and 50% PBS) overnight at 4°C. Cells were then washed with 0.1% Tween 20 and incubated a mixture of Goat anti-Rabbit IRDye 800 (CW) (Cat# 926–32202, RRID: AB_2883971, 1:800 dilution, Li-Cor, Lincoln, USA) and Cell-Tag 700 total protein reagent (1:500 dilution, Li-Cor, Lincoln, USA) for 1 h at room temperature. After washing with 0.1% Tween 20, the plates were scanned on an Odyssey Infrared Imager (Li-Cor) using the 96-well plate scanning template option according to the manufacturer’s instructions. The 800-channel intensity representing PLVAP protein detection was normalised for the 700-channel Cell-Tag signal using the Li-Cor Odyssey software to control for variations in cell number and well-to-well detection efficiency.

### 2.5 | Western blotting

HRMECs were cultured in 60 mm Petri dishes (4 × 10^5^). Membrane proteins from whole cell lysates were extracted with the subcellular protein fractionation kit for cultured cells (Cat# 78840, Thermo Fisher, Waltham, MA, USA). Total protein content was determined by means of the BCA assay kit (Pierce^™^ BCA Protein Assay Kit, Invitrogen, Life Technologies, Carlsbad, USA). Extracted proteins (20 μg) were loaded on 4–12% Tris–glycine gel. After electrophoresis, proteins were transferred into a nitrocellulose membrane (Invitrogen, Life Technologies, Carlsbad, CA). Membranes were blocked with milk, 5% Tris buffered saline, and 0.2% Tween 20 (TBST) for 1 h at room temperature. Membranes were incubated overnight (4°C) with appropriate primary anti-PLVAP (mouse, 1:500 dilution in TBST, Cat# ab81719, RRID:AB_1658370, Abcam, Cambridge, UK), anti- P2X7 receptor (rabbit, 1:500 dilution in TBST-5%BSA, Cat# 13809, RRID: AB_2798319, Cell Signaling Technology, Danvers, MA, USA) and anti-β-Actin (rabbit, 1:1000 dilution in TBST, Cat# A2066, RRID:AB_476693, Sigma-Aldrich, St Louis, MO) antibodies. After overnight incubation, the membranes were then incubated with secondary chemiluminescent antibody (ECL anti-mouse, Cat# NA931, RRID:AB_772210 and ECL anti-rabbit, Cat# NA934, RRID:AB_772206, 1:2000 dilution, Cytiva Amersham, United Kingdom) for 1 h at room temperature. After secondary antibody, the membranes were incubated with ECL (SuperSignal^™^ West Pico PLUS Chemiluminescent Substrate, Thermo Fisher Scientific, Carlsbad, CA, USA) and were detected through I-Bright^™^ 1500 (Invitrogen, Life Technologies, Carlsbad, CA, United States) by using chemiluminescence. Densitometry analyses of blots were performed at non-saturating exposures and analysed using ImageJ software (NIH, Bethesda, MD). Values were normalised to β-actin, which was also used as loading control. These immunoblot protocols are compatible with the relevant *British Journal of Pharmacology* Editorial (Alexander et al., 2018).

### 2.6 | In vivo experimental design

The ethical review board of Vasile Goldiş Western University of Arad approved the animal experimental protocols (Authorisation no. 135/1.03.2019), ensuring adherence to European and national regulations concerning laboratory animal research. Additionally, the in vivo experimental protocols adhered with the Association for Research in Vision and Ophthalmology (ARVO) Statement for the Use of Animals in Ophthalmic and Vision Research, in compliance with the ARRIVE guidelines (Percie du Sert et al., 2020) and with the recommendations made by the *British Journal of Pharmacology* (Lilley et al., 2020).

Male CD1 mice (Balta et al., 2024; Trotta et al., 2023), weighing between 28 and 30 g, obtained from Vasile Goldiş Western University’s animal unit, were selected for this study, and we considered only male mice, based on the findings showing that microvascular complications may affect prevalently diabetic males (Maric-Bilkan, 2017). They were housed under conditions that conformed to established national and European regulatory requirements. To induce diabetes, a single i.p. injection of streptozotocin (STZ; 102 mg kg^−1^; Daniels Gatward et al., 2021) was used, resembling aspects of early phases of diabetic retinopathy, and specifically to refine the experimental model and to reduce number of animals (Balta et al., 2024; Trotta et al., 2023). This solution of STZ was freshly constituted in a citrate buffer (50 mM, pH 4.5). Fasting (4 h) blood glucose levels were monitored (one-touch glucometer, Accu Chek Active, Roche Diagnostics, USA) 24 and 48 h post-STZ injection, and then at 2 weeks post-STZ administration, identifying hyperglycaemic conditions as levels exceeding 2 mg ml^−1^. Mice that met this criterion were considered diabetic and included in the study. The cut-off value of 15% of weight loss was fixed as exclusion criteria for the experimental procedure, and weight was monitored during the treatment protocol. Diabetic mice (*n* = 20) were randomly assigned to two experimental group: sterile PBS intravitreal injection group (*n* = 10) and to P2X7 receptor antagonist treatment group (*n* = 10). No animals were excluded during the experimental protocol, on grounds of significant weight loss or signs of discomfort, because of diabetes. Specifically, diabetic mice (*n* = 10) received 2 μl of sterile PBS intravitreal injection at intervals of 2, 6 and 10 weeks, subsequent to the diabetes assessment and were killed (by cervical dislocation), 12 weeks after the experiment’s onset. In a parallel cohort, designated as the JNJ group (*n* = 10), intravitreal injections of the P2X7 receptor antagonist (JNJ47965567) (2 μl of 300 nM) were carried out at specific time points (Figure 1) (Gesualdo et al., 2021; Rossi et al., 2016). This dose has been chosen in order to attain a JNJ47965567 concentration of 100 nM in the mouse vitreous, taking into account the vitreous volume (Bhattacharya et al., 2013; Fresta, Caruso, et al., 2020; Kaplan et al., 2010; Platania et al., 2017, 2019). The control mice (*n* = 10) were subjected to citrate buffer (50 mM, pH 4.5) i.p. injection and then received intravitreal sterile PBS (2 μl), as their diabetic counterparts. Before intravitreal injections, mice were anaesthetised through intraperitoneal injection of ketamine-xylazine (100–10 mg kg^−1^). Subsequently, ocular anaesthesia was obtained through administration of 1% tetracaine eyedrops (5 μl each eye). To obtain pupil dilatation mice received 5% tropicamide (5 μl each eye). The JNJ47965567 solution was prepared freshly in accordance with the provider’s guidelines immediately before its use. The mice were killed at 12 weeks by cervical dislocation, and their eyes were immediately enucleated and processed for subsequent analyses.

### 2.7 | Immunodetection in vitro and in vivo

ZO-1 immunodetection was carried out as follows. Glass chamber slides were coated with a fibronectin (1 mg ml^−1^) for 1 h at 37°C and washed with sterile water. HRMECs (6 × 10^4^ cells well^−1^) were seeded on 24-well fibronectin coated glass chamber slides. Cells were incubated for 5 days at 37°C in a 5% CO_2_ humidified atmosphere. Cell adhesion and confluence was reached within 5 days, and the medium was changed every 2 days. Cells were shifted for 1 h with medium containing JNJ47965567 (100 nM) and for 48 h to a medium containing 40 mM glucose with JNJ47965567 (100 nM; HG + JNJ group of cells). HRMECs growth in medium with physiological glucose concentration (5 mM) served as control cells (Ctrl), and HRMECs growth in high glucose (40 mM, 48 h) corresponded to positive control group (HG). After 48 h, cells were fixed with ice-cold acetone for 15 min and with ice-cold methanol for 20 min. Thereafter, cells were washed with cold phosphate buffered saline (PBS, pH 7.4) and blocked with 5% normal goat serum (NGS) and 0.1% Triton X-100 in PBS solution, for 30 min at room temperature. Cells were then incubated overnight at 4°C with primary antibody against ZO-1 (rabbit, Cat# 61–7300, RRID: AB_2533938, dilution 1:100, Thermo Fisher Scientific, Monza, Italy). After overnight incubation and primary antibody washout with PBS, the secondary anti-rabbit Alexa 488-coniugated antibody (dilution 1:200 in TBST, Cat# ab96899, RRID: AB_10679361, Abcam, Cambridge, UK) was added for 1 h at room temperature in the dark. VE-cadherin immunodetection was carried out with a different protocol. HRMECs (6 × 10^4^ cells well^−1^) were seeded on 24-well fibronectin coated glass chamber slides pre-coated with fibronectin (1 mg ml^−1^) for 1 h at 37°C and then incubated for 5 days at 37°C in a 5% CO_2_ humidified atmosphere. The medium was changed every 2 days. Thereafter, the cells were transferred to a different medium, as described for ZO-1 staining. After 48 h of treatment, the cells were fixed with 4% paraformaldehyde for 15 min at room temperature, washed twice with cold PBS and permeabilised with 0.3% Triton X-100 in PBS (pH 7.4) for 5 min at room temperature. After blocking with 1% bovine serum albumin (BSA) in PBS for 1 h, the cells were incubated with the rabbit anti-VE-cadherin antibody (rabbit, dilution 1:100, Cat# 2500, RRID:AB_10839118, Cell Signalling, Technology, Danvers, MA, USA) in 1% BSA–PBS solution, overnight at 4°C. Then, the slides were washed three times with PBS, and 1 h incubation was carried out with anti-rabbit Alexa 488-coniugated secondary antibody (1:200 dilution, Cat# ab96899, RRID:AB_10679361, Abcam, Cambridge, UK) at room temperature in the dark. After washing again, the slides were mounted using Fluoroshield^™^ with DAPI (Cat# F6057–29ML Sigma-Aldrich, St Louis, MO). Images were acquired by using the Zeiss Observer Z1 microscope (Carl Zeiss Microscopy GmbH, Oberkochen, Germany). A semi-quantitative evaluation of the ZO-1 and Ve-cadherin localisation at cell–cell interface was carried out by analysing images from slides of each condition (*n* = 6). The images (*n* = 6 per group) were analysed, and ImageJ was used for measurements of the average grey scale.

HRMECs (6 × 10^4^ cells well^−1^) were seeded on 24-well fibronectin coated glass chamber slides pre-coated with fibronectin (1 mg ml^−1^) for 1 h at 37°C and then incubated for 5 days at 37°C in a 5% CO_2_ humidified atmosphere. Cell adhesion and confluence was reached within 5 days, and the medium was changed every 2 days. Also in this case, three experimental groups were used for this experiment: Ctrl (cells growth in 5-mM glucose medium for 48 h); HG (cells growth in 40-mM glucose medium for 48 h); HG + JNJ (cells pre-treated for 1 h with 100 nM JNJ47965567 and then transferred to 40-mM glucose medium with 100 nM JNJ47965567). After 48 h of treatment, the cells were fixed with 2% paraformaldehyde for 30 min at 4°C, washed twice with cold PBS and permeabilised with 0.2% Triton X-100 in PBS (pH 7.4) for 5 min at 4°C. After blocking with 5% NGS in PBS for 1 h, the cells were incubated with the rabbit anti-VE-cadherin and anti-PLVAP antibodies in 1% BSA–PBS solution, overnight at 4°C. After overnight incubation, the slides were washed three times with PBS. Then, 1 h incubation was carried out with anti-mouse IgG H + L (DyLight 550) secondary antibody in 0.1% Triton X-100 in PBS (1:300 dilution, Cat# 115-165-062, RRID:AB_2338685, Jackson ImmunoResearch Labs, Cambridge, UK) and with anti-rabbit Alexa 488-coniugated secondary antibody (1:200 dilution, Cat# ab96899, RRID:AB_10679361, Abcam, Cambridge, UK) at room temperature in the dark. Nuclei staining was carried out with Fluoroshield^™^ with DAPI (Cat# F6057–29ML Sigma-Aldrich, St Louis, MO). Finally, the slides were mounted using mounting medium (Life Technologies, Monza, Italy). Images were acquired with a fluorescence microscope Zeiss Observer Z1 equipped with the Apotome.2 acquisition system connected to a digital camera (Carl Zeiss, Oberkochen, Germany). A semi-quantitative evaluation of the PLVAP and VE-cadherin colocalisation at the cell–cell interface was carried out analysing images from slides of each condition (*n* = 6). The images (*n* = 6 per group) were analysed, and ImageJ was used for measurements.

Immunofluorescence analysis was performed on paraffin-embedded eye sections (*n* = 10). Particularly, the eyes were fixed in 10% neutral-buffered formalin for 24 h, followed by dehydration through increasing ethanol concentrations (70%, 80%, 90% and 100%), clearing in xylene and embedding in paraffin. Tissue sections, 5-μm thick, were cut and mounted on glass slides. The paraffin embedded slides were deparaffinised and rehydrated in an alcohol gradient (100%, 96% and 70% volume). After washing, the antigen unmasking was performed with sodium citrate buffer (pH 6.0). Slides were blocked with 1% BSA and 5% normal goat serum in PBS solution, washed with PBS and incubated with a rat anti-mouse monoclonal PLVAP antibody (Cat# MCA2539, RRID:AB_931736, Bio-Rad, USA) and AlexaFluor 546 labelled goat anti-rat IgG secondary antibody (Cat# A-11081, RRID: AB_2534125, Thermo Fisher Scientific Inc., Rockford, IL, USA). Bond Dewax Solution (Leica Biosystems Inc., Buffalo Grove, IL, USA) was used to deparaffinate the eye sections. They were rehydrated in alcoholic solutions. Epitope Retrieval Solution (Leica Biosystems Inc., Buffalo Grove, IL, United States) was used for antigen retrieval at 95°C for 10 min, followed by blocking with 2% BSA in PBS. The primary antibody was applied in a dilution of 1:50 in primary antibody diluting buffer (Bio-Optica, Milano, Italy) for 2 h at 4°C. The slides were washed in PBS and incubated with the secondary antibody, diluted to 1:500 in PBS, for 2 h at room temperature in the dark. After a further three washing steps with PBS, nucleus counterstaining was performed with 1 μg ml^−1^ DAPI (Sigma-Aldrich, St Louis, MO, USA). CC/Mount aqueous mounting medium (Sigma-Aldrich, St Louis, MO, USA) was used to mount the stained slides. They were examined with a Leica SP5 confocal microscope. These immunostaining protocols adhered to the relevant *British Journal of Pharmacology* editorial (Alexander et al., 2018). Image J 6.4 software was utilised to carry out a semi-quantitative analysis of PLVAP immunostaining. The process included quantification of fluorescence across various sections. For each section assessed, five arbitrary retina fields were selected for measurement. The data obtained were then expressed as a percentage reflecting the fluorescence intensity.

### 2.8 | Data and statistical analysis

Investigators were unaware of experimental group labels, which were unveiled after graph design and statistical analysis, that was carried out by another investigator. In this study, data analysis complied with the recommendations on experimental design and analysis in pharmacology as reported in the *British Journal of Pharmacology* Editorial (Curtis et al., 2022). Data shown are means ± SD. We carried out *n* = 6 independent in vitro experiments, and each sample was run in triplicate. Data from in vivo studies are reported as mean ± SD, *n* = 10 animals per group; we carried out immunostaining analyses of 10 eyes per group, and five fields were selected randomly from each eye section. Sample size has been chosen after power analysis, setting *P* <0.05 as the cut-off value. After analysis of normal distribution of data through the Shapiro–Wilk test, one-way ANOVA, followed by Tukey–Kramer post hoc test, was carried out for multiple comparisons. Post hoc tests were carried out when *F* was *P* < 0.05, and no significant variance inhomogeneity was found within groups. Differences between groups were considered significant at *P* < 0.05. Statistical analyses were carried out with SPSS software, version 21.0 (SPSS Inc., Chicago, IL, USA), and GraphPad Prism version 10 (San Diego, CA, USA); this latter software has been used for graph design.

### 2.9 | Materials

JNJ47965567 was supplied by Tocris Bioscience (Bristol, UK) and streptozotocin (U-9889, CAS 18883-66-4) by Santa Cruz Biotechnology (Dallas, TX, USA).

### 2.10 | Nomenclature of targets and ligands

Key protein targets and ligands in this article are hyperlinked to corresponding entries in http://www.guidetopharmacology.org, and are permanently archived in the Concise Guide to PHARMACOLOGY 2021/2022 (Alexander, Fabbro et al., 2023; Alexander, Mathie et al., 2023; Di Virgilio et al., 2023).

## 3 | RESULTS

### 3.1 | Gene network analysis

Our experimental hypothesis was based on a putative link between P2X7 receptor activity and PLVAP expression, leading to detrimental effects on barrier integrity, because of transcellular transport promoted by PLVAP itself. In this context, based on our previous work and other published work, we explored the hypothesis that P2X7 receptor and PLVAP belong to the same gene–gene or protein–protein network, at first through a computational approach (Figure 2) and then experimentally (Figures 3 and 4). The webserver STRING predicted the following P2X7 receptor interactors in the protein–protein interaction network (Figure 2 and Table S1): the inflammasome NLRP3, IL-1β, connexin 43 (GJA1), VEGF-A, PIGF, TSPAN12, LRP5, ZO-1, VE-cadherin and PLVAP. K-means clustering showed that P2X7 receptor signalling (including the inflammasome and IL-1β) forms a cluster, directly connected to the largest cluster that includes angiogenic factors (VEGF-A and PIGF) along with proteins involved in biological barrier stability (ZO-1, VE-cadherin, GJA1 and PLVAP). TSPAN12 and LRP5 formed an isolated cluster connected only with VEGF-A (Figure 2).

### 3.2 | P2X7 receptor antagonist counteracts expression of angiogenic and inflammatory markers in retinal endothelial cells

The treatment with JNJ47965567 did not affect viability of primary human pericytes (Platania et al., 2017), and of HRMECs (data not shown). We confirmed, with experimental approaches, the connections (shown in Figure 2) between P2X7 receptor activation and expression of proteins involved in angiogenesis, inflammation and BBB stability in diabetic retinopathy. First, we carried out qPCR on mRNA extracts from primary retinal endothelial cells, challenged with high glucose levels (40 mM), with or without exposure to a P2X7 receptor antagonist (JNJ47965567, 100 nM) (Figures 3 and 4).

High glucose levels increased, in retinal endothelial cells, levels of VEGF-A, PIGF, P2X7 receptor and IL-1β (Figure 3). No significant changes in TSPAN12 and LRP5 mRNA levels were detected in HRMECs challenged with 40 mM glucose (HG), compared to control cells. Block of the P2X7 receptor, with 100 nM JNJ47965567, reversed the effects of HG. Thus, VEGF-A, PIGF, P2X7 receptor and IL-1β mRNA levels were decreased after treatment with JNJ47965567. The expression of TSPAN12 and LRP5 was downregulated by the P2X7 receptor antagonist, compared to expression in control cells and in the cells exposed to high glucose levels (Figure 3).

### 3.3 | P2X7 receptor antagonist preserves BRB integrity

As regards the expression of genes encoding for proteins directly involved in the stability of the BRB, we found that high glucose levels increased the expression of PLVAP, and decreased the expression of ZO-1, VE-cadherin and connexin-43, compared to control cells (Figure 4). This expression pattern was modified by the treatment with JNJ47965567 (Figure 4). Specifically, the P2X7 receptor antagonist counteracted the effects of high glucose concentration in retinal endothelial cells, with PLVAP mRNA levels decreasing and, conversely, levels of mRNA for ZO-1, VE-cadherin and connexin 43 increasing (Figure 4).

To confirm the effect of JNJ47965567 on BRB integrity, we also analysed the cell membrane localisation of tight junction (TJ) proteins, in particular of ZO-1 and VE-cadherin, by immunocytochemistry (Figure 5). As already observed for mRNA levels, high glucose levels led to an abnormal expression of both proteins in the HRMECs monolayers, compared to control cells (Figure 5). However, pre-treatment with JNJ47965567 protected HRMECs from HG-damage, preventing the reduction of ZO-1 and VE-cadherin expression induced by 48 h of exposure to HG (Figure 5), and restoring the proper localisation of these key inner BRB (iBRB) proteins.

### 3.4 | P2X7 receptor antagonist regulates PLVAP expression

To evaluate if the protein expression of P2X7 receptor and PLVAP was in line with observed mRNA modulation (Figures 3e and 4a), we employed western blot analysis. P2X7 receptor protein overexpression, induced by 40 mM glucose, was prevented by the P2X7 receptor antagonist (Figure 6a). Pre-treatment of retinal endothelial cells with JNJ47965567, followed by exposure to high glucose levels, induced a significant reduction of PLVAP protein expression, as demonstrated by in-cell western blot technique (Figure 6b) and standard western blot (Figure 6c). Considering that PLVAP dimers are stabilised in the cell membrane by extensive N-glycosylation, we aimed also to assess PLVAP glycosylation level induced by high glucose. High glucose levels promoted PLVAP glycosylation, while exposure to JNJ47965567 decreased glycosylated PLVAP levels to control cell values (Figure 6d).

Under physiological conditions, PLVAP protein is not expressed in the endothelium which forms the BBB and BRB, so its expression represents an index of BBB and BRB breakdown. For this reason, we aimed at evaluating the protein expression, and also its localisation in BRB, in relation to VE-cadherin (Figures 7 and 8). Through semi-quantitative analysis of immunofluorescence, we assessed the loss of integrity of BRB induced by high glucose treatment, which led to overexpression of PLVAP protein at cytoplasm and cell–cell interface. Treatment with JNJ47965567 restored the integrity of the BRB, stimulating expression of the junction protein VE-cadherin and reducing expression of PLVAP, not only in the cytoplasmic region but also, in particular, at the cell–cell interface (Figures 7 and 8).

### 3.5 | PLVAP expression is modulated by the P2X7 receptor antagonist in mice with diabetic choroidopathy

Immunostaining with antibody targeting PLVAP in diabetic mice retina revealed pronounced labelling within the fenestrated endothelial cells of the choriocapillaris, which frequently seemed to have a double layer adjacent to their retinal pigmented epithelium (RPE) side (Figure 9). Conversely, after treatment with JNJ47965567, PLVAP staining intensity was diminished in these ocular structures, aligning closely with that of the control group (Figure 9).

### 3.6 | P2X7 receptor enriched interaction network

Genes found to be modulated in our model of diabetic retinopathy were the input of an enrichment analysis carried out with STRING application on Cytoscape (v. 3.7.0). The number of interactors was extended to 10 and 20, applying a 0.4 confidence score. Enrichment data have been loaded. We show, in Figure 10, the network representation obtained extending the hypothetical interactors to 10, because network parameters and connectivity was not significantly modified in the predicted network. Inspecting Figure 10a, it is clear that three proteins (PLVAP, P2X7 receptor and PIGF) showed the highest value of clustering coefficient and neighbourhood connectivity. In contrast, Figure 10b showed that PLVAP, P2X7R and PIGF had the lowest value of betweenness centrality, meaning that these proteins are not crossed by shortest paths and are involved in secondary pathways within the analysed network. Nevertheless, if we consider the proteins that have the highest betweenness value, another cluster, consisting of eight proteins, emerged (in order CTNNB1, VEGFA, KDR, CDH5, AKT1, ACTB, PECAM1 and GJA1). The betweenness value of each of these proteins is greater than the average betweenness value of the entire network. Each protein in this cluster plays a ‘bridging’ role in the network and each of the eight proteins is crossed by a large number of shortest paths. Indeed, we are in the presence of two dichotomous node clusters, with two distinct dual properties.

## 4 | DISCUSSION

Diabetic retinopathy is characterised by microvascular lesions with pericyte loss and endothelial cell death, also during the early stages of the disease. The inflammatory and oxidative events lead to necrosis and apoptosis of vascular cells. In particular, the elevated levels of ATP are strictly related to the activation and overexpression of P2X7 receptors, leading to chronic inflammation. PLVAP, an endothelial-specific protein, is widely expressed in capillaries and venous vasculature, during pathological conditions, and correlates with expression of VEGF-A activating the VEGFR2 signalling pathway (Bosma et al., 2018; Van Der Wijk et al., 2019; Wisniewska-Kruk et al., 2016).

We found that high glucose levels promoted the expression of pro-angiogenic factors (PIGF, VEGF) in retinal endothelial cells, along with dysregulation of P2X7 receptor and connexin 43 expression, as noted earlier (Lazzara et al., 2019; Platania et al., 2019), and the expression of these genes was decreased by treatment with the P2X7 receptor antagonist. Moreover, we found that block of the P2X7 receptor preserved the barrier integrity, decreasing the expression of PLVAP, along with increased expression of adherens and tight junctions, as previously reported (Fresta, Caruso, et al., 2020; Fresta, Fidilio, et al., 2020; Lazzara et al., 2022). In our in vitro model, blockade of P2X7 receptor reversed these effects and decreased the expression of two pro-angiogenic factors - tetraspanin 12 (TSPAN12) and LDL receptor-related protein 5 (LRP5) (Bucher et al., 2017; Wang et al., 2022; Xu et al., 2022), which belong to the Norrin signalling pathway, that is itself essential for the developmental of the retinal vasculature and the BRB (Zuercher et al., 2012). High barrier endothelia of the CNS express very low levels of PLVAP, compared to lower barrier endothelia elsewhere in the body. In the case of the mouse neural retina, formation of the functional BRB is not fully complete with the formation of tight junctions, but later when expression of PLVAP is diminished (Van Der Wijk et al., 2019). A major factor that suppresses PLVAP expression during retinal vascular development is the Norrin–Wnt signalling pathway. In fact, additional expression of LRP5 and TSPAN12 form a Norrin-sensitive binding complex comprised of FZD_4_/LRP5/TSPAN12 (Wang et al., 2018), and inhibition of Norrin–Wnt signalling results in increased PLVAP expression (Zhang et al., 2018). In our study, while LRP5 and TSPAN12 gene expression was not perturbed over 48 h by high glucose, the block of P2X7 receptor activity partly decreased the expression of both genes, suggesting that P2X7 receptor signalling may have some role, direct or indirect, in the unique expression of these two genes in retinal endothelial cells.

Looking at PLVAP itself, a substantial increase in expression of the gene and protein was found after 48 h exposure to high glucose, damaging the integrity of the endothelial cell monolayer. The effect of P2X7 receptor blockade was a marked decrease in PLVAP expression, which had been promoted by high glucose exposure. Additionally, the P2X7 receptor antagonist JNJ47965567 decreased significantly the expression of glycosylated PLVAP in primary microvascular endothelial cells. N-glycosylation of PLVAP, near the transmembrane domain, is essential for stabilisation of PLVAP supramolecular aggregates (wheel shape) in diaphragms, fenestrae and trans-endothelial channels (TECs) (Chang et al., 2023; Stan et al., 1999). Our results show, for the first time, that high glucose increased glycosylated PLVAP levels in microvascular endothelial cells, stabilising PLVAP supramolecular structures, which in turn promote trans-endothelial and endothelial permeability. Antagonism of the P2X7 receptors almost abolished N-glycosylation of PLVAP, inhibiting endothelial permeability. This suggests that P2X7 receptor signalling is a pathway with marked effects on the regulation of PLVAP gene expression, transduction, post-transcriptional modification and aggregation, in addition to its effects on VEGF-A signalling. On the basis of our results, we cannot exactly establish the contribution of VEGF-A and PIGF gene expression, both elicited by high glucose, in driving PLVAP expression. However, the elevated expression of both genes was also blocked by the P2X7 receptor antagonist. Furthermore, we evaluated PLVAP expression and its localisation in an iBRB model (retinal endothelial cells monolayer), in relation to VE-cadherin protein. We demonstrated that the treatment with JNJ47965567 restored iBRB integrity, stimulating the expression of the junction protein and reducing the amount of PLVAP, in particular at cell–cell interface, according to previous findings regarding the key role of PLVAP in BRB integrity (Wisniewska-Kruk et al., 2016).

A limitation of our study is related to the use of retinal endothelial cell monolayer (2D model) instead of a model including also retinal pericytes, astrocytes (Fresta, Fidilio, et al., 2020; Maurissen et al., 2024) or Müller cells (Mazzeo et al., 2020). However, although a holistic approach, including more than one cell type or providing transcriptome and/or proteome information (Lazzara et al., 2024; Quinn et al., 2024), would address the complexity inherent in a biological system, the in vitro reductionist approach we have used has allowed the direct analysis of the link between P2X7 receptors and PLVAP. Importantly, this finding was corroborated by the results in our in vivo model of diabetic retinopathy.

In our in vivo model, we demonstrated that administration of P2X7 receptor antagonist to mice decreased PLVAP labelling in retinal choriocapillaris of mice with diabetic choroidopathy. We were able to see the expression of PLVAP, and its negative modulation by P2X7 receptor antagonist, in mouse choroid. Involvement of the choroid in our in vivo model of diabetic retinopathy is not surprising, because earlier and more recent studies have highlighted the finding that choroidal vessels are affected in patients with diabetic retinopathy. Thus, choroidal pathology in diabetic retinopathy was first described in 1969 (Yanoff, 1969) and defined as diabetic choroidopathy in 1985 (Hidayat & Fine, 1985). However, diabetic choroidopathy is infrequently diagnosed in clinical practice, because of limited access to specific diagnostic techniques and instruments. Recently, optical coherence tomography angiography (OCTA) from both the retinal and choroidal microvasculature has been used to detect the presence and severity of diabetic retinopathy (Tan et al., 2023). In terms of the PLVAP expression in the choroid, our results are congruent with findings highlighting that the Norrin/Wnt pathway is indirectly involved in choroidal vessels (Seitz et al., 2018). We were not able to see PLVAP staining in iBRB vessels, probably for different reasons. Specifically, iBRB vessels are not fenestrated, as are the choroidal vessels (Le et al., 2023). More likely, we missed the timeframe in which PLVAP is expressed in endothelial cells of iBRB of diabetic mice. This is one of our study’s limits, which may be covered with further investigations, in particular by analysis of PLVAP expression in the retina of diabetic mice, before 12 weeks of diabetes.

We demonstrated, for the first time, that P2X7 receptor signalling was tightly linked also to PLVAP expression in both in vitro and in vivo models of diabetic retinopathy used in this study. As mentioned by Bosma et al. (2018) and Denzer et al. (2023), regulation of PLVAP expression appears to be strictly related to VEGFR signalling (Nakagami et al., 2019), along with other inflammatory cytokines. But, on the basis of our results, we cannot attribute the decrease in PLVAP expression induced by P2X7 receptor blockade, to inhibition of VEGFR or of Norrin signalling pathways. However, a holistic approach based on network analysis on our experimental data helped the interpretation of results.

The preliminary network, based on published data, showed a direct link between P2X7 receptor and the inflammasome, in agreement with experimental data showing a strong link between P2X7 receptors and NPLR3 inflammasomes (Kong et al., 2022). Additionally, analysis of the experimentally built network showed that P2X7 receptors, PLVAP and PIGF are part of secondary paths and do not play a ‘bridging’ role in the network. They are, however, peripheral, almost isolated, nodes of the network. Thus these gene products may act as switches in the pathology of diabetic retinopathy and, interestingly, P2X7 receptor have been shown to act as a switch in inflammation and cell death signalling (Ho et al., 2016).

The ‘bridging’ role in the network is played by the gene products with the highest betweenness value (i.e., in order, CTNNB1 and VEGF-A). Thus, these gene products represent essential nodes in the network structure and wiring. VEGF-A is the target of currently approved intravitreal drugs for treatment of proliferative diabetic retinopathy. However, these anti-VEGF agents are pan-VEGF inhibitors (Varey et al., 2008). CTNNB1 encodes for β-catenin and represents the downstream effector of the Wnt signalling pathway, which was found to be aberrant in in vivo models of diabetic retinopathy and also in retinal sections from patients with diabetic retinopathy (Chen & Ma, 2017). Although VEGF-A and Wnt signalling are essentially involved in pathogenic mechanisms of diabetic retinopathy or retinal neovascularisation, these pathways may have an hormetic function, and patients treated with drugs targeting these pathways would encounter drug resistance and/or neurodegeneration secondary to treatment (Apte et al., 2019). In this context, on the basis of the structure of the network, we can hypothesise that P2X7 receptor signalling effects on PLVAP is indirect, through modulation of both VEGFR and β catenin signalling pathways.

In conclusion, these findings enrich the complex roles played by P2X7 receptors in diabetic retinopathy. Among the already evaluated pathways, we have here demonstrated that P2X7 receptor action also modulated PLVAP protein, together regulating the integrity of the endothelial barrier. Apart from VEGF-A and PLVAP, P2X7 receptors also regulate the expression of PIGF, another important pro-angiogenic factor.

Drugs modulating P2X7 receptors, PLVAP and PIGF may provide innovative treatments for diabetic retinopathy because of their isolated role in the expression network of genes involved in this condition.

## Supplementary Material

Supplemental Table and Figure

Additional supporting information can be found online in the Supporting Information section at the end of this article.

## Figures and Tables

**FIGURE 1:**
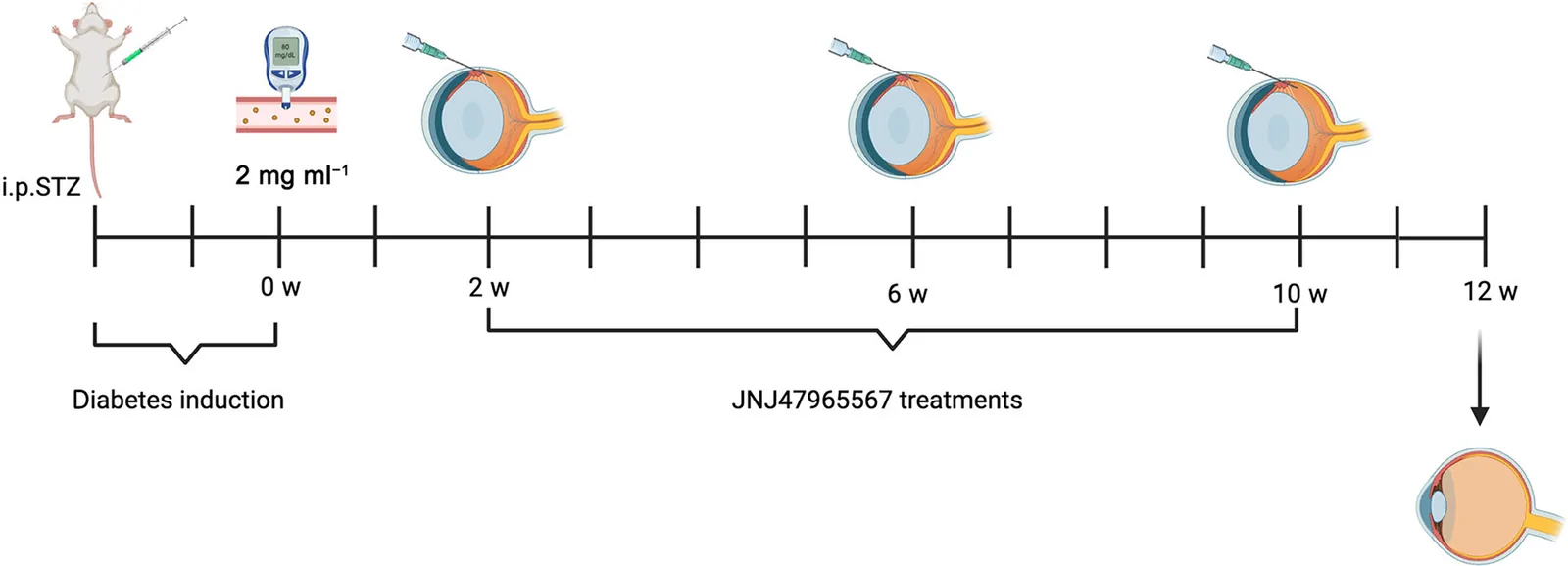
In vivo study design. Male CD1 mice received a single intraperitoneal (i.p.) injection of streptozotocin (STZ, 102 mg kg) to induce diabetes. Two weeks later, diabetes was assessed by hyperglycaemic levels exceeding 2 mg ml^−1^ (0w). Diabetic mice received intravitreal injections of JNJ47965567 (2 μl of 300 nM) or PBS (2 μl) at 2, 6 and 10 weeks after diabetes assessment (respectively, 2 w, 6 w and 10 w). Animals were killed after 12 weeks from 0 w (12 w), and eyes were enucleated for subsequent analysis. Figure created with BioRender.com.

**FIGURE 2:**
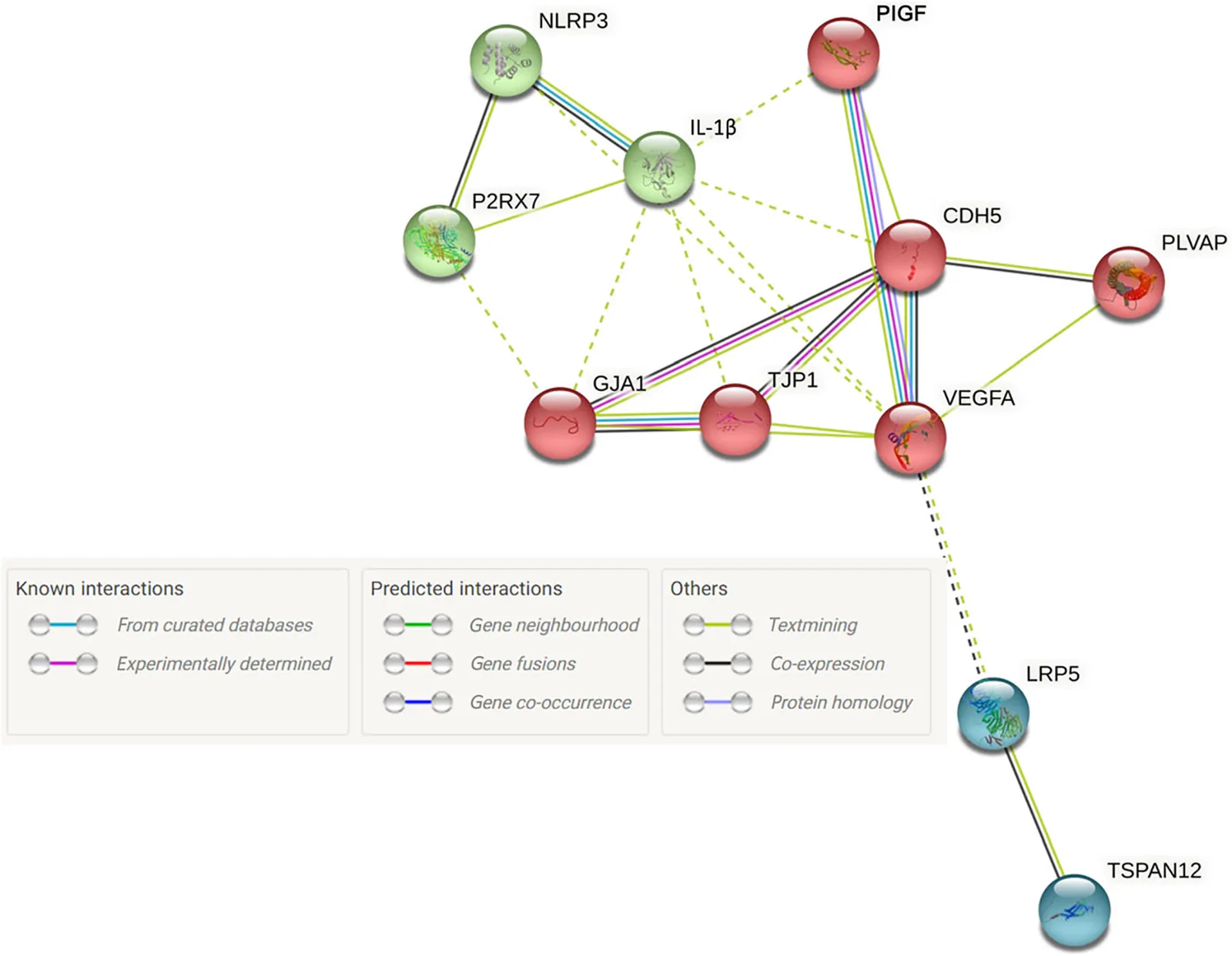
Predicted P2X7 regulatory network. P2RX7 (P2X7 receptor), NLRP3 (inflammasome), IL-1β, GJA1 (connexin 43), TJP1 (ZO-1), VEGF-A, CDH5 (VE-cadherin), PIGF (placental growth factor), PLVAP (plasmalemma-associated protein), TSPAN12 (tetraspanin 12) and LRP5 (LDL receptor-related protein 5).

**FIGURE 3:**
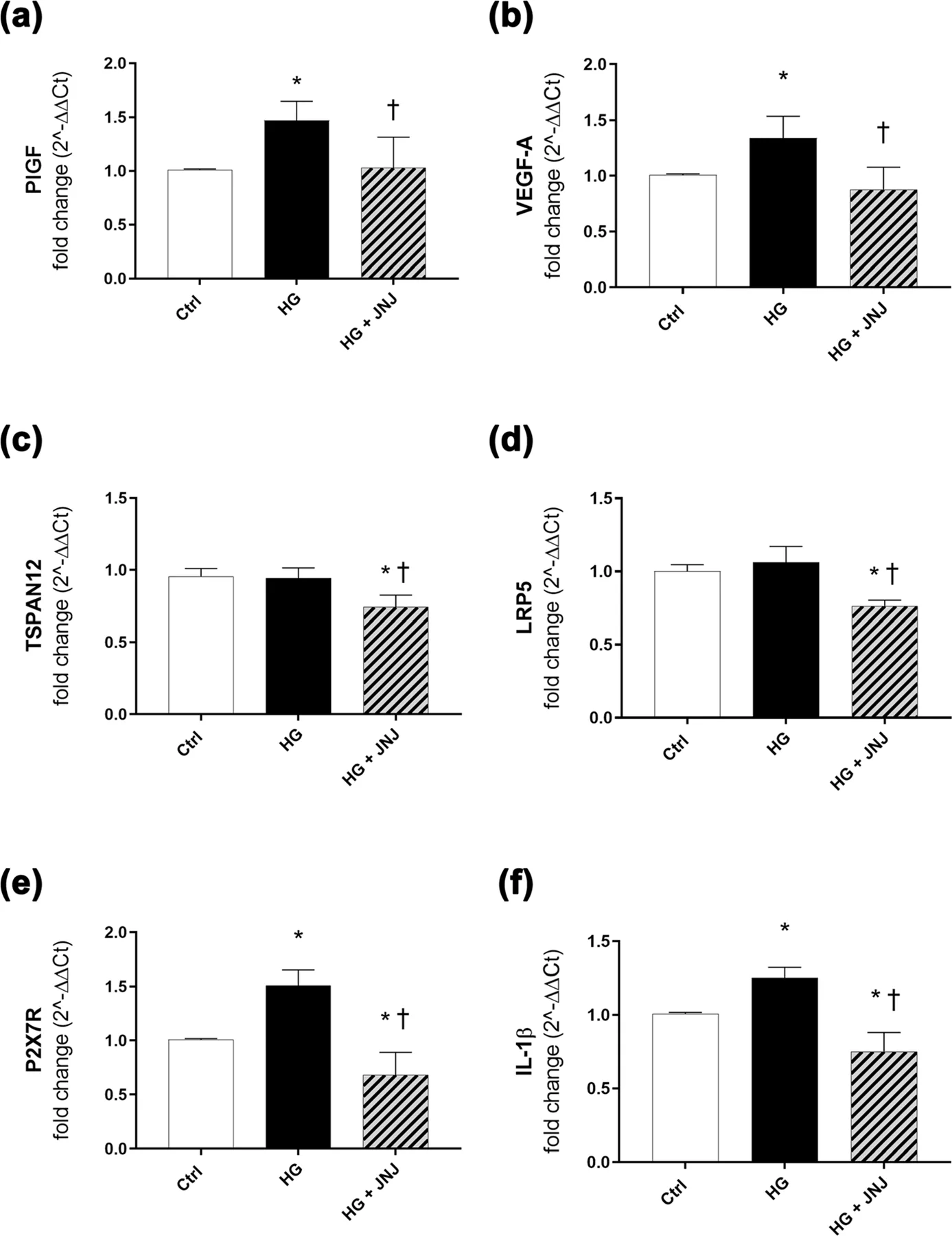
Gene-expression profile of proteins involved in angiogenesis and inflammation. mRNA levels of (a) PIGF, (b) VEGF-A, (c) TSPAN12, (d) LRP5, (e) P2X7 receptor (P2X7R) and (f) IL-1β in microvascular retinal endothelial cells, pretreated with JNJ47965567 (JNJ, 100 nM) for 1 hour and subsequently co-treated with high glucose (HG, 40 mM) for 48 h. The mRNA levels were evaluated by qPCR. Data shown are means ± SD (n = 6; each run in triplicate). *P <0.05, significantly different from control; †P<0.05, significantly different from HG; one-way ANOVA and Tukey post -hoc test for multiple comparisons.

**FIGURE 4:**
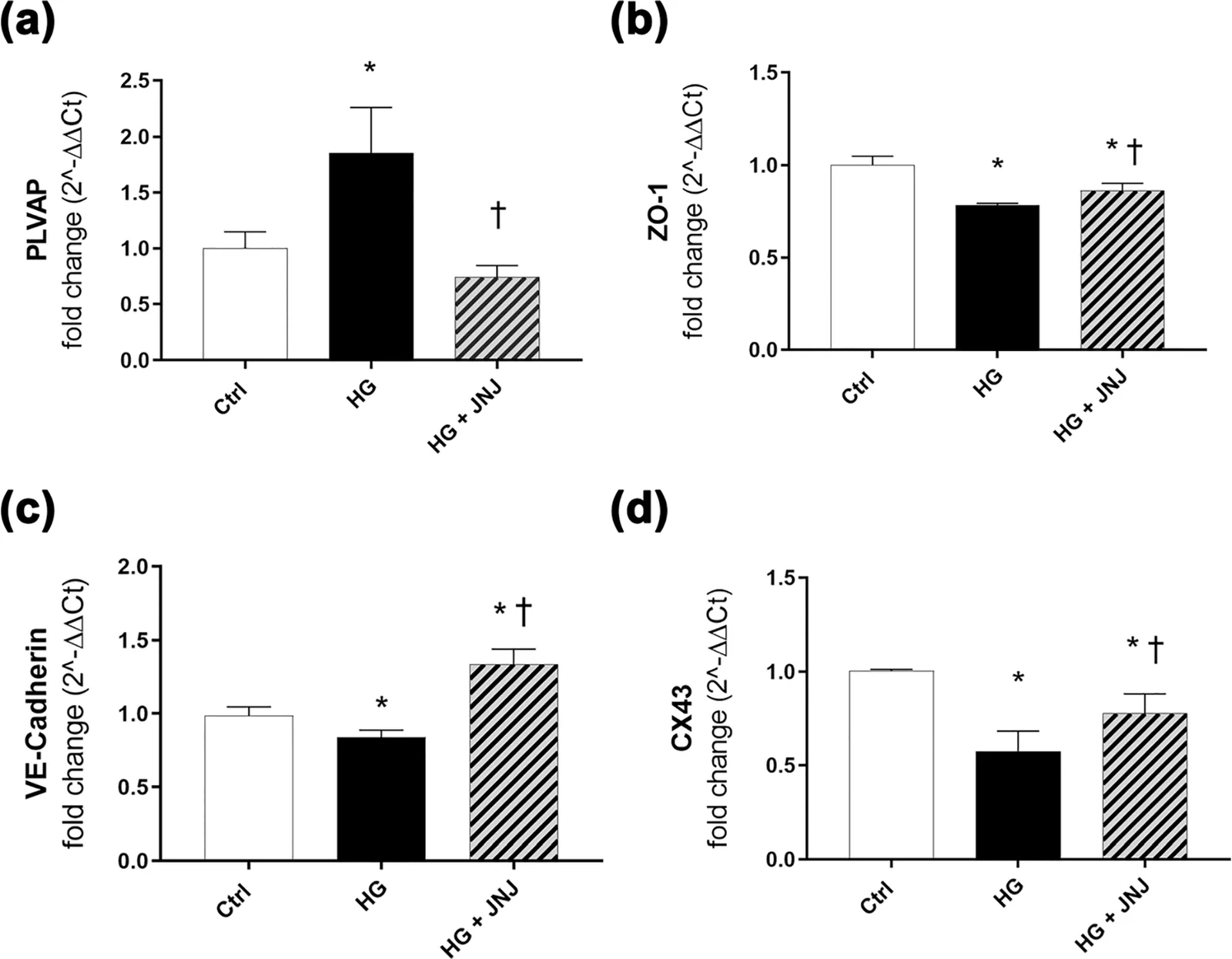
Gene-expression profile of proteins involved in BRB stability. mRNA levels of (a) PLVAP, (b) tight- (ZO-1), (c) adherens- (VE-Cadherin, CHD5) and (d) GAP-junctions (connexin 43, CX43 or GJA1) in retinal endothelial cells, pretreated with the P2X7 receptor antagonist JNJ47965567 (100 nM) for 1 hour and subsequently co-treated with high glucose (HG, 40 mM) for 48 h. The mRNA levels were evaluated by qPCR. Data shown are means ± SD; n = 6; each run in triplicate. *P<0.05, significantly different from control; †*P*< 0.05, significantly different from HG; one-way ANOVA and Tukey post -hoc test for multiple comparisons.

**FIGURE 5:**
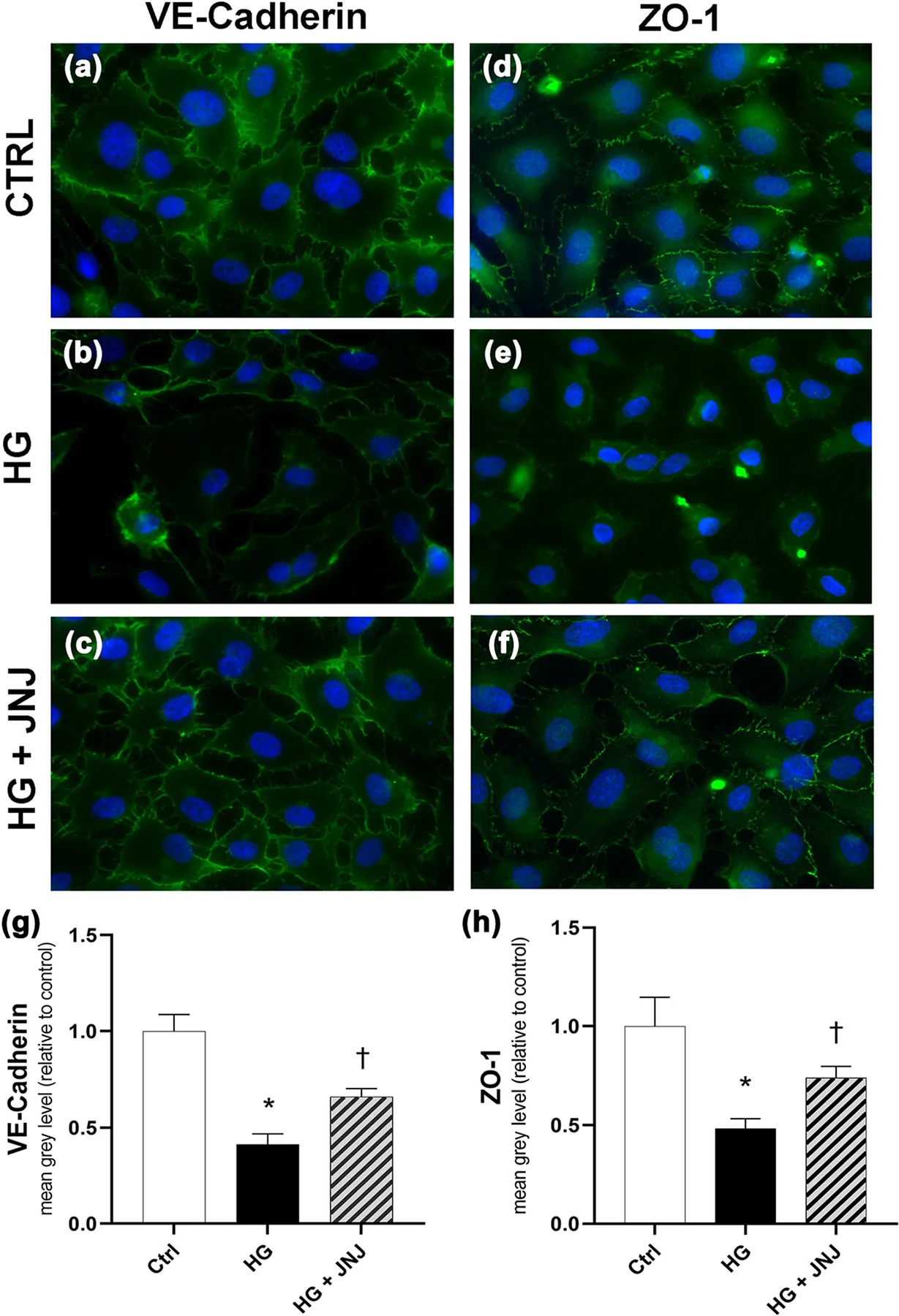
The P2X7 receptor antagonist maintains iBRB integrity through modulation of VE-cadherin and ZO-1 expression. HRMECs were pretreated with JNJ47965567 (JNJ, 100 nM) for 1 hour and subsequently co-treated with high glucose (HG, 40 mM) for 48 h. Representative images for (a–c) VE-cadherin and (d–f) ZO-1 localization in HRMECs after treatment with HG and JNJ. VE-cadherin and ZO-1 were labelled with FITC (green); nuclei were labelled with DAPI (blue). Images were acquired at 40X magnification. Scale bar: 10 μm. Fluorescence semi-quantification of (g) VE-cadherin and (h) ZO-1 protein (mean grey levels) at cell–cell interface. Data shown are means ± SD; n = 6. *P < 0.05, significantly different from control; †*P* < 0.05, significantly different from HG; one-way ANOVA and Tukey post -hoc test for multiple comparisons. Semi-quantitative analyses have been carried out with ImageJ.

**FIGURE 6:**
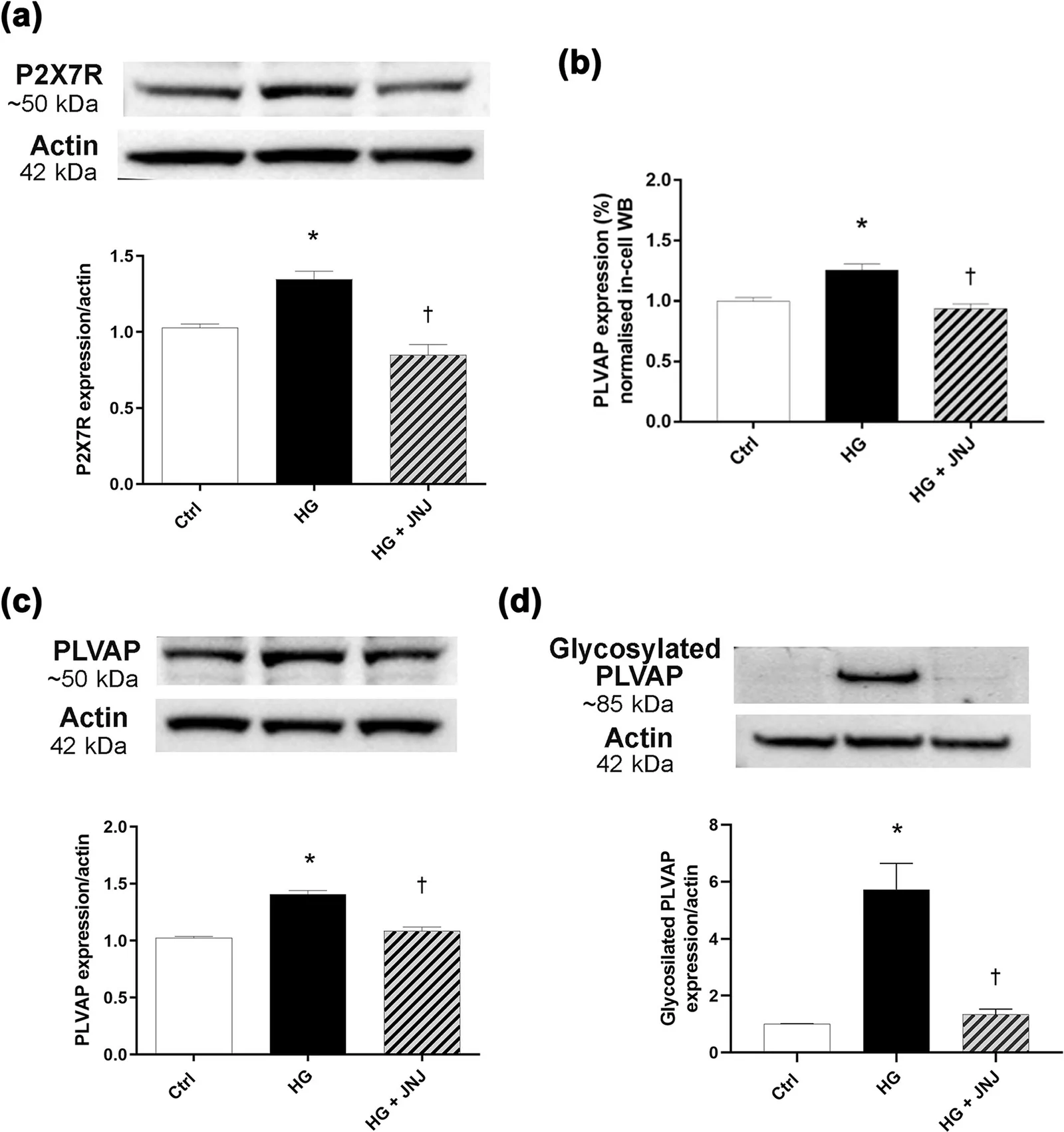
The P2X7 receptor antagonist JNJ47965567prevents expression of P2X7 receptor and PLVAP protein. Western blot analysis. Representative blots of PLVAP and P2X7 receptor proteins extracted from HRMECs cells pre-treated for 1 h with JNJ47965567 (JNJ, 100 nM) and subsequently co-treated with high glucose (HG, 40 mM) for 48 h. (a) P2X7 receptor (P2X7R), (c) PLVAP, (d) glycosylated PLVAP. Densitometry analysis of each band was carried out with the Image J programme. Data shown are means ± SD; (*n* = 6; each run in triplicate). (b) In-cell Western analysis (in situ) of PLVAP. *P < 0.05, significantly different from control; †*P* < 0.05, significantly different from HG; one-way ANOVA and Tukey post -hoc test for multiple comparisons.

**FIGURE 7:**
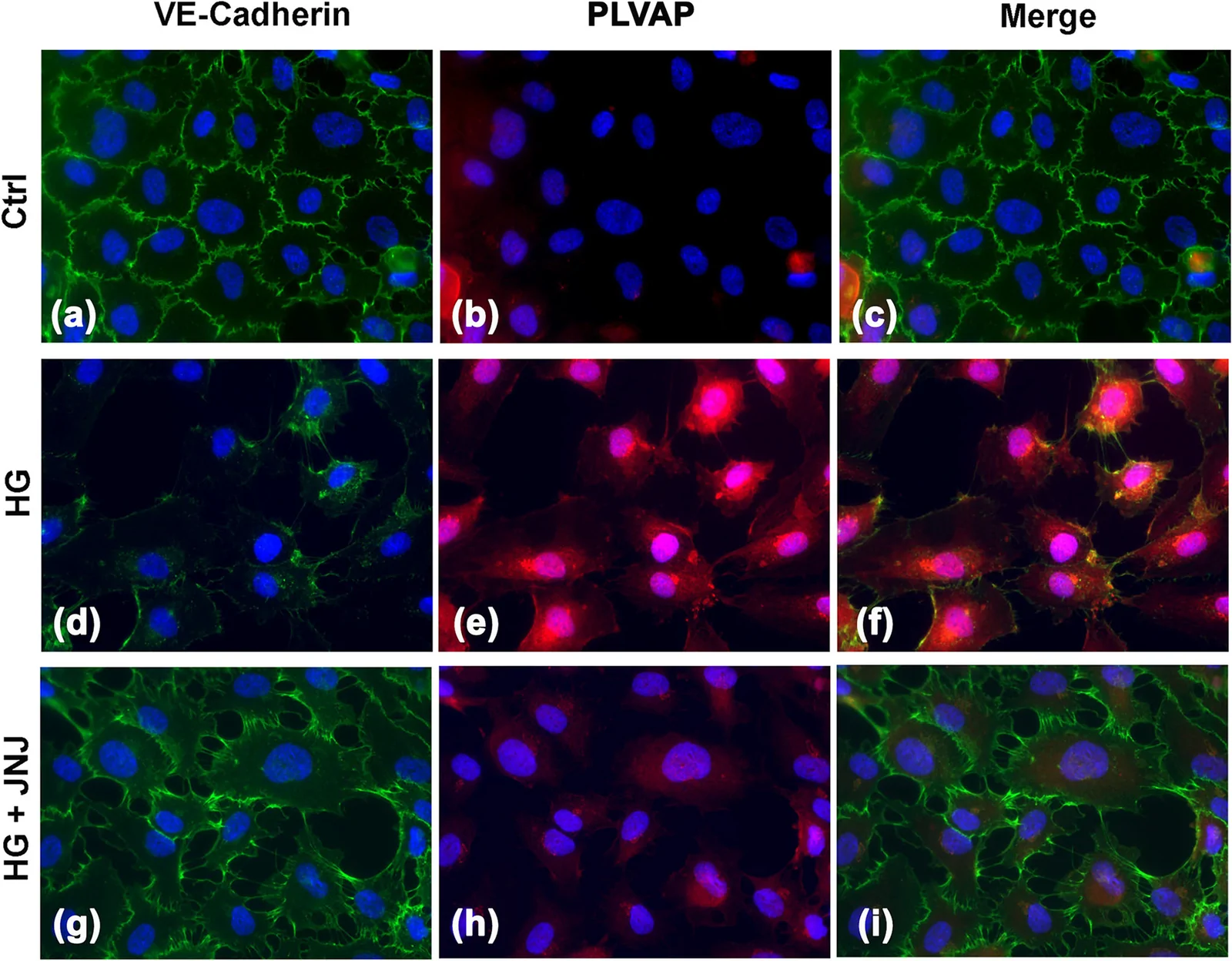
Effects of P2X7 receptor antagonist, JNJ47965567, on VE-cadherin and PLVAP expression in HG-challenged HRMECs. Representative images of double immunofluorescence staining with antibodies against PLVAP (red) and the endothelial cell junction protein, VE-cadherin (green). (a–c) Control cells (Ctrl) were characterised by membrane localisation of VE-cadherin and negligible PLVAP expression. (d–f) Treatment with HG (40 mM) for 48 h induced the loss of VE-cadherin expression and localisation, together with a significant citosolic and membrane expression of PLVAP protein. The pre-treatment with JNJ47965567 (JNJ) induced a recovery in VE-cadherin expression and localisation, with a significant reduction of PLVAP expression (g–i). Nuclei were labelled with DAPI (blue). Images were acquired at 40X magnification. Scale bar: 10 μm. (*n* = 6).

**FIGURE 8:**
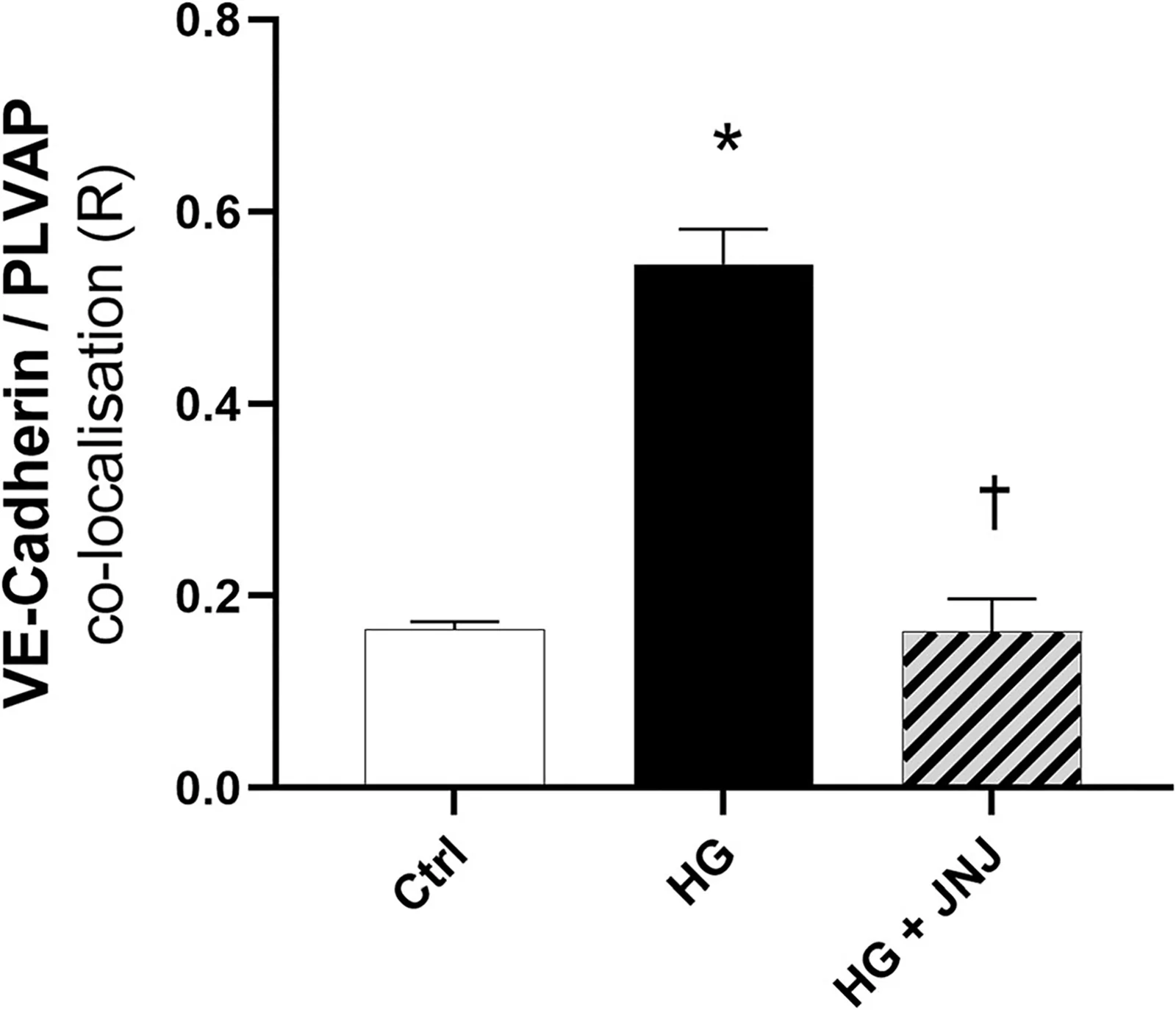
Co-localisation of VE-cadherin and PLVAP. High glucose (HG) promoted PLVAP expression and its localisation at cell membrane (VE-cadherin/PLVAP co-localisation). The P2X7 receptor antagonist, JNJ47965567 (JNJ) abolished PLVAP expression at the cell membrane. R, Pearson correlation coefficient for VE-cadherin and PLVAP expression. (*n* = 6). *P < 0.05, significantly different from control (Ctrl); †*P* < 0.05 significantly different from HG; semi-quantitative analyses have been carried out with ImageJ.

**FIGURE 9:**
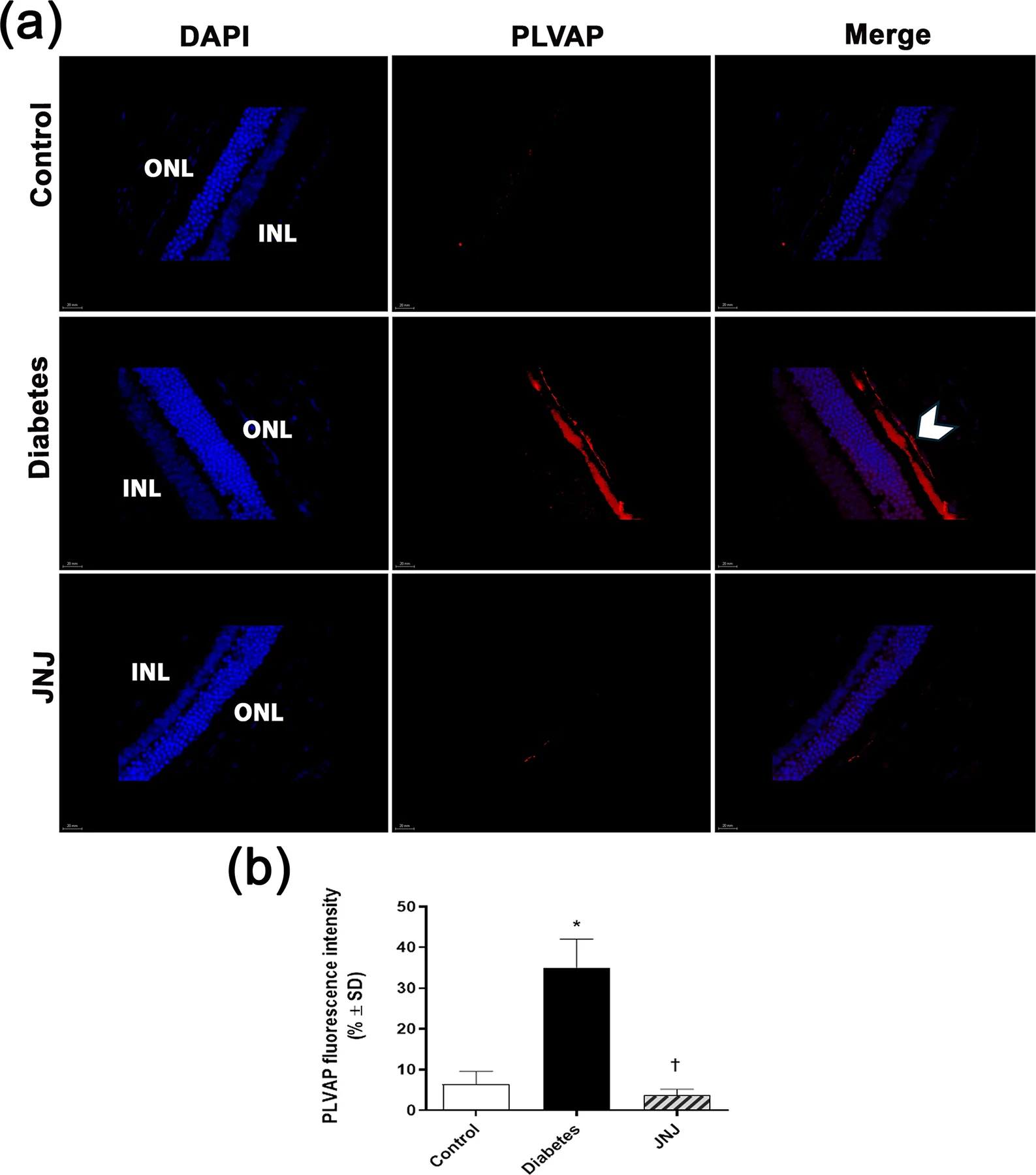
Effects of P2X7 receptor antagonist (JNJ) on PLVAP expression in the choriocapillaris of mice with diabetic choroidopathy. (a) Immunolabelling with antibody against PLVAP (red). Nuclei were labelled with DAPI (blue). Images were acquired at 40X magnification at a Leica SP5 confocal microscope. Outer nuclear layer (ONL); inner nuclear layer (INL); arrowhead labelling the fenestrated endothelial cells of the choriocapillaris which appeared to be double-layered on their RPE side (n = 10 eyes per group). (b) Bar graphs showing semi-quantification of fluorescence intensity for PLVAP in retina, as means ± SD. *P < 0.05, significantly different from control; †*P* < 0.05, significantly different from diabetes. Semi-quantitative analyses have been carried out with ImageJ.

**FIGURE 10:**
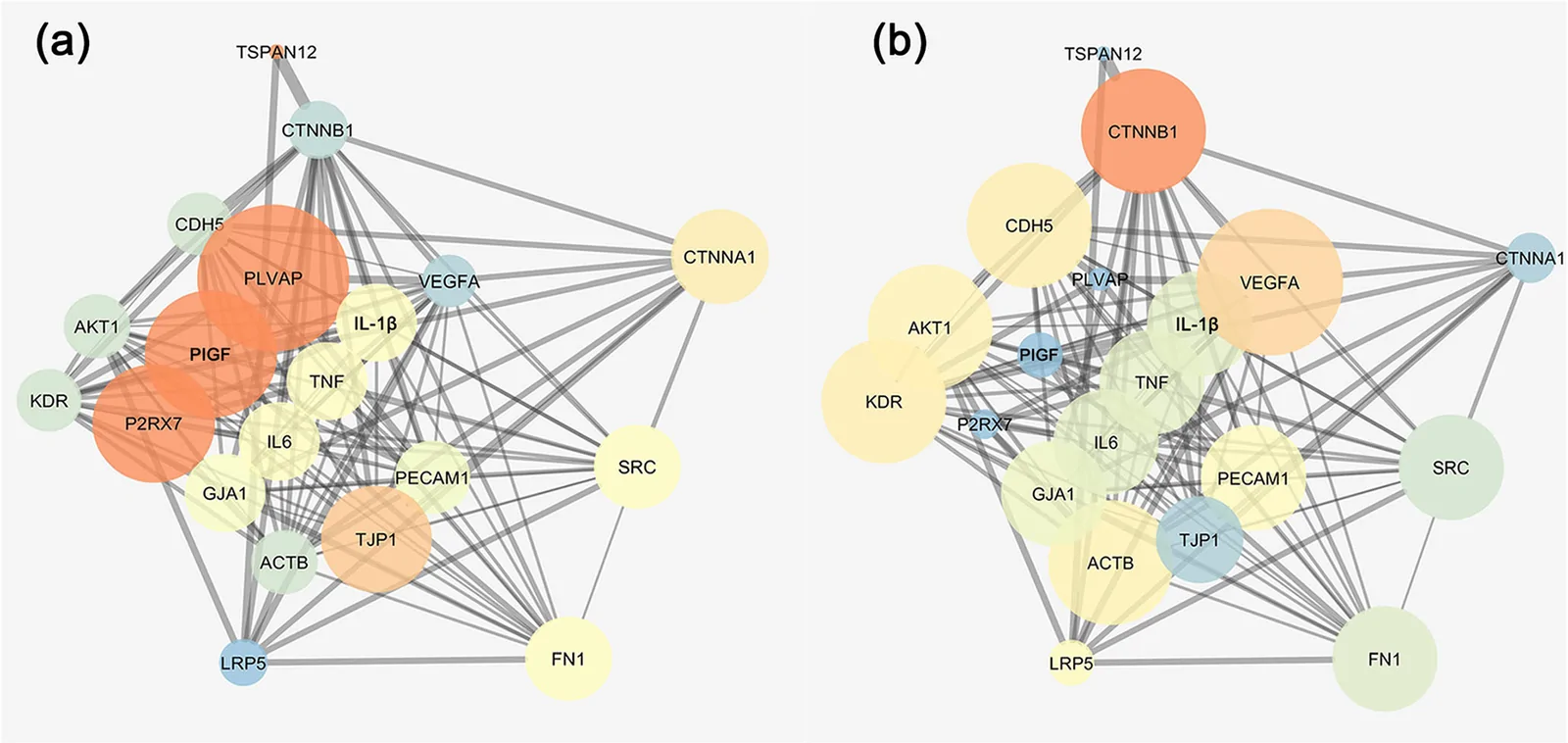
Enriched interaction network based on experimental data. (a) Node dimension is proportional to clustering coefficient, while neighbourhood connectivity is mapped as b-factor (blue to red, low to high values). (b) Node dimension is proportional to closeness centrality values, while betweenness centrality is mapped as b-factor (blue to red, low to high values).

## Data Availability

The data that support the findings of this study are available from the corresponding author upon reasonable request. Some data may not be available because of privacy or ethical restrictions.

## What is already known?

- Expression of plasmalemma vesicle-associated protein (PLVAP) is increased during pathological conditions.

## What does this study add?

- P2X7 receptor antagonist blocked overexpression of PLVAP and maintained BRB integrity in high glucose conditions.

## What is the clinical significance?

- Inhibition of P2X7R/PLVAP pathway could be useful to treat patients affected by diabetic retinopathy.

## Abbreviations:

BRB: blood-retinal barrier
CHD5: cadherin 5
GJA1: gap junction protein alpha 1
HG: high glucose
HRMECs: primary human retinal microvascular endothelial cells
LRP5: LDL receptor-related protein 5
PIGF: placental growth factor
PLVAP: plasmalemma vesicle-associated protein
ROP: retinopathy of prematurity
STRING: search tool for the retrieval of interacting genes/proteins
TSPAN12: tetraspanin 12
VEGF-A: vascular endothelial growth factor-A
ZO-1: zonula occludens-1
