# Supplementary material for: Blockade of P2X7 receptors preserves blood retinal barrier integrity by modulating the plasmalemma vesicle-associated protein: Implications for diabetic retinopathy
Source: Br J Pharmacol. Author manuscript; Available in PMC 2026 Nov 9. (PMC13746355; doi:10.1111/bph.70007)
Supplement: Supplementary file 1 [file NIHMS2127487-supplement-Supplemental_Table_and_Figure.pdf]

Purinergic P2X7 receptor antagonism preserves blood retinal barrier integrity decreasing plasmalemma vesicle associated protein expression: implications for diabetic retinopathy

Chiara Bianca Maria Platania[1,2†], Francesca Lazzara[1,2†], Kenneth Mitton[3], Naomi Haque[3], Wendelin Dailey[3], Federica Conti[1], Erika Giuffrida[1], Filippo Drago[1,2], Anca Hermenean [4], Cornel Balta [4], Hildegard Herman [4], Alina Ciceu [4], Maria Consiglia Trotta[5], Michele D'Amico[5], Giuseppe Nicosia[1], Settimio Rossi[6] and Claudio Bucolo[1,2*]

[1]Department of Biomedical and Biotechnological Sciences, School of Medicine, University of Catania, Catania, Italy; [2]Center for Research in Ocular Pharmacology —CERFO, University of Catania, Catania, Italy; [3]Eye Research Institute, Oakland University, Rochester, MI, USA; [4] Aurel Ardelean" Institute of Life Sciences, Vasile Goldis Western University of Arad, Arad, Romania [5] Department of Experimental Medicine, Division of Pharmacology, University of Campania "Luigi Vanvitelli", Naples, Italy; [6] Eye Clinic, Multidisciplinary Department of Medical, Surgical and Dental Sciences, University of Campania "Luigi Vanvitelli", Naples, Italy;

[†]*co-first*

[*]*Corresponding author: claudio.bucolo@unict.it*

Table S1. Functional Enrichment Analysis

#term ID	Term description	Strength	False discovery rate	Matching proteins in your network (labels)
GO:0035633	Maintenance of blood-brain barrier	2.32	2.92e-05	TJP1, GJA1, CDH5, VEGFA
GO:0045765	Regulation of angiogenesis	1.55	2.92e-05	TSPAN12, IL-1β, TJP1, CDH5, PGF, VEGFA
GO:0045766	Positive regulation of angiogenesis	1.72	4.12e-05	IL-1β, TJP1, CDH5, PGF, VEGFA
GO:0001894	Tissue homeostasis	1.61	0.00011	TJP1, GJA1, P2RX7, CDH5, VEGFA
GO:0030334	Regulation of cell migration	1.16	0.00011	PLVAP, IL-1β, TJP1, GJA1, CDH5, PGF, VEGFA
GO:1903140	Regulation of establishment of endothelial barrier	2.55	0.00011	IL-1β, CDH5, VEGFA
GO:0030335	Positive regulation of cell migration	1.31	0.00012	PLVAP, IL-1β, TJP1, CDH5, PGF, VEGFA
GO:0007165	Signal transduction	0.6	0.00015	TSPAN12, PLVAP, IL-1β, TJP1, GJA1, LRP5, P2RX7, NLRP3, CDH5, PGF, VEGFA
GO:0007267	Cell-cell signalling	1.04	0.00029	TSPAN12, IL-1β, GJA1, LRP5, P2RX7, NLRP3, PGF
GO:0043114	Regulation of vascular permeability	2.07	0.00083	TJP1, CDH5, VEGFA

GO:0002711	Positive regulation of t cell mediated immunity	2.06	0.00091	IL-1β, P2RX7, NLRP3
GO:0003018	Vascular process in circulatory system	1.58	0.00091	TJP1, GJA1, CDH5, VEGFA
GO:1901888	Regulation of cell junction assembly	1.55	0.0011	IL-1β, TJP1, GJA1, VEGFA
GO:0001568	Blood vessel development	1.25	0.0014	TSPAN12, LRP5, CDH5, PGF, VEGFA
GO:0002684	Positive regulation of immune system process	1.05	0.0015	PLVAP, IL-1β, P2RX7, NLRP3, PGF, VEGFA
GO:0007166	Cell surface receptor signalling pathway	0.79	0.0015	TSPAN12, PLVAP, IL-1β, LRP5, P2RX7, CDH5, PGF, VEGFA
GO:0042981	Regulation of apoptotic process	0.9	0.0015	IL-1β, GJA1, LRP5, P2RX7, NLRP3,CDH5,VEGFA
GO:0071310	Cellular response to organic substance	0.78	0.0015	PLVAP, IL-1β, GJA1, P2RX7, NLRP3, CDH5, PGF, VEGFA
GO:0032101	Regulation of response to external stimulus	1.02	0.0018	IL-1β, GJA1, NLRP3, CDH5, PGF, VEGFA
GO:0042592	Homeostatic process	0.87	0.0020	IL-1β, TJP1, GJA1, LRP5, P2RX7, CDH5, VEGFA
GO:0060754	Positive regulation of mast cell chemotaxis	2.77	0.0020	PGF, VEGFA
GO:1902396	Protein localization to bicellular tight junction	2.77	0.0020	TJP1, CDH5
GO:1905603	Regulation of blood-brain barrier permeability	2.77	0.0020	TJP1, VEGFA
GO:0032308	Positive regulation of prostaglandin secretion	2.55	0.0039	IL-1β, P2RX7
GO:0032496	Response to lipopolysaccharide	1.36	0.0042	IL-1β, GJA1, P2RX7, NLRP3
GO:0032611	interleukin-1beta production	2.38	0.0066	IL-1β, NLRP3

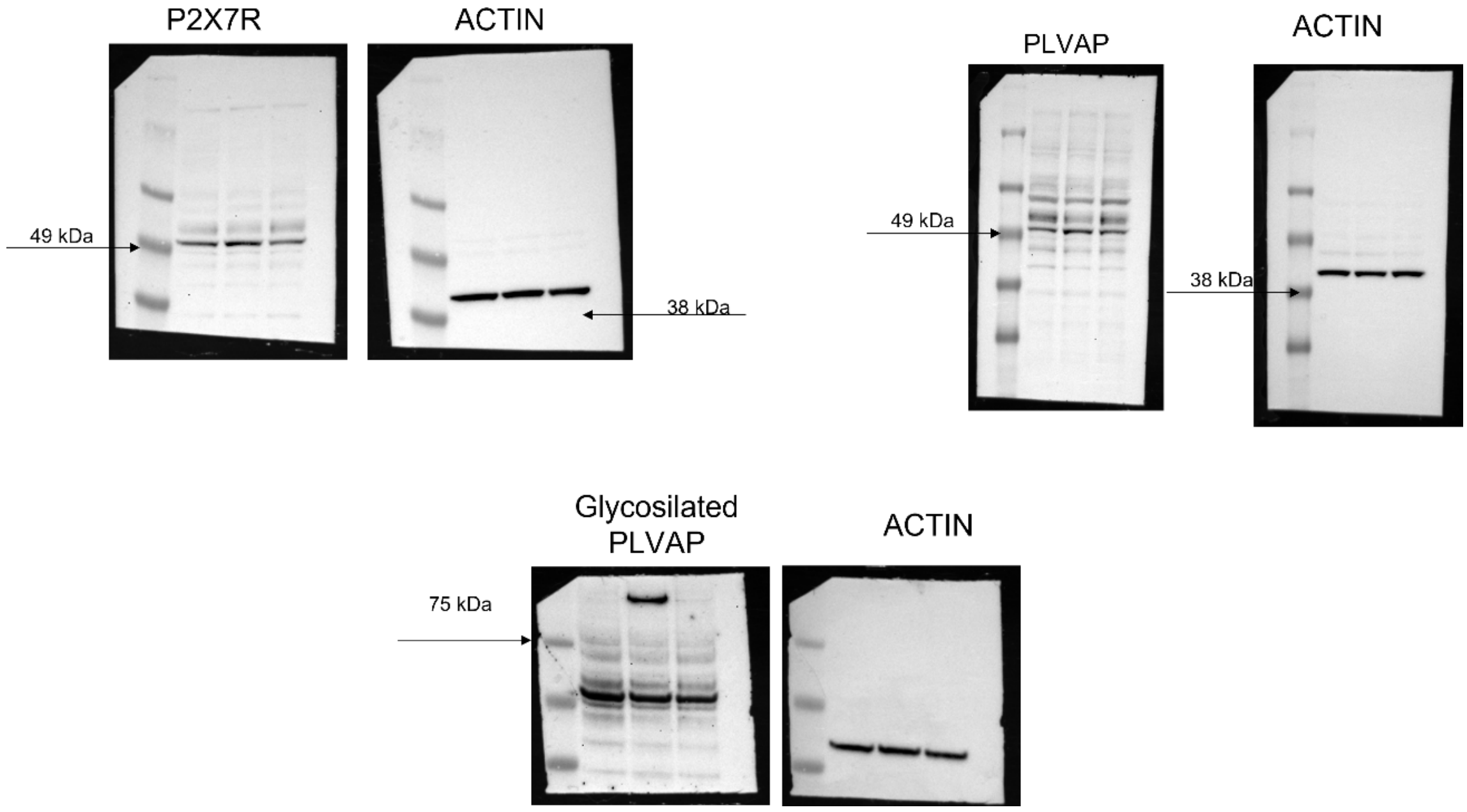

Figure S1: Full scan (uncropped) western blots of figure 6. Molecular weight markers are indicated.
